# Books and Magazines

**Published:** 1905-02

**Authors:** 


### Books and Magazines.

REGIONAL MINOR SURGERY. By George Gray Van Schaick, Consulting Surgeon to French Hospital, New York. Second edition, enlarged and revised, 228 pages, bound in cloth, profusely illustrated. Price, $1.50. International Journal of Surgery Co., New York.

The practicability and usefulness of this book is best indicated by the demand, necessitating a second edition in an unusual short time. This edition has been subjected to a thorough revision and additional chapters have been added.

PNEUMONIA AND PNEUMOCOCCUS INFECTION. By Robert B. Preble, A. B., M. D., Professor of Medicine, Northwestern University. Illustrated. Chicago: Cloyd J. Head & Co. 1905. Price, $1.00.

This book is a summary of the advances that have been made in our knowledge of this the most fatal of diseases, and is a fair exposition of the up-to-date teachings and treatment.

Messrs. Lea Brothers & Co. have pleasure in announcing for early publication a completely new work which will be welcomed by every practitioner, teacher and student. Hare's Practice of Medicine, a text-book of the practice of medicine. For students and Practitioners. By Hobart Amory Hare, M. D., B. S., Professor of Therapeutics and Materia Medica in the Jefferson Medical College of Philadelphia; Physician to the Jefferson Medical

College Hospital; Laureate of the Royal Academy of Medicine in Belgium, of the Medical Society of London. Author of A Text-Book of Practical Diagnosis, etc. In one very handsome volume of about 1000 pages, with about 100 engravings and six full-page plates in colors and monochrome.

---

ATLAS AND EPITOME OF DISEASES OF THE MOUTH, PHARYNX, AND NOSE. By Dr. L. Grunwald, of Munich. From the second revised and enlarged German edition. Edited, with additions, by James E. Newcomb, M. D., Instructor in Laryngology, Cornell University, Medical School; Attending Laryngologist to the Roosevelt Hospital, Out-Patient Department. With 102 illustrations on 42 colored lithographic plates, 41 text-cuts, and 219 pages of text. Philadelphia and London: W. B. Saunders & Co., 1903. Cloth, $3.00 net.

In designing this atlas the author has kept constantly in mind the needs of both student and practitioner, and as far as possible, typical cases of the various diseases have been selected. The illustrations are described in the text in exactly the same way as a practiced examiner would demonstrate the objective findings to his class, the book thus serving as a substitute for actual clinical work. The illustrations themselves are numerous and exceedingly well executed, portraying the conditions so strikingly that their study is almost equal to examination of the actual specimens. The editor has incorporated his own valuable experience, and has also included extensive notes on the use of the active principle of the suprarenal bodies in the materia medica of rhinology and laryngology. The work, besides being an excellent atlas and epitome of the diseases of the mouth, pharynx, and nose, serves as a text-book on the anatomy and physiology of these organs. Indeed, we wonder how the author has encompassed so much within such a limited space. We heartily commend the work as the best we have seen.

---

NEW METHODS OF TREATMENT. By Dr. Laumonier. Translated and edited from the second revised and enlarged French edition by H. W. Syers, M. A., M. D. (Cantab.), Chicago. 1904. W. T. Keener & Co. Cloth; 316 pages; $2.50.

This book is rather a work on new medicinal remedies than on new methods of treatment. The author says that recently "no striking discovery has been effected and sanctioned by experience in the domain of therapeutics. Hence, here will merely be found in addition (to last edition) some articles devoted to *adrenalin,* to *salicylate of methyl,* to *ulmarene,* to *quinoformine,* to *collargol,* and to the *colliod metals* which have lately been much discussed."

### THE ONE REMEDY

**which experience proves is free from detrimental effects is**

### GRAY'S Glycerine TONIC Comp.

**Try it in convalescence, respiratory disorders, anaemia, malnutrition, nervous exhaustion.**

**THE PURDUE FREDERICK CO.,**
**298 Broadway, New York**

He treats of certain ferments recently experimented on; namely, *oxydoses, reductases* and *kinases*, and says that the remarkable properties of these ferments may perhaps be attributed to the traces of radio-active substances which they contain.

---

Normal Histology and Microscopical Anatomy. Jeremiah S. Ferguson, M. Sc., M. D., Instructor in Histology, Cornell University Medical College, New York. 462 illustrations; many in color. D. Appleton & Co., New York. Cloth, $4.00. Sold only by subscription.

This work is believed to be the very best and most extensive upon this subject ever published in English. Many subjects are exhaustively treated that are hardly mentioned in most text-books on the subject. Of special note is the section upon the central nervous system and the organs of special sense. The illustrations are excellent, and are worthily made from original drawings. The *Journal* very highly commends it to student and practitioner alike.

---

The Practical Medicine Series. Year-Book Publishing Company, Chicago. The series consists of ten volumes, published monthly, and covers the entire field of medicine and surgery.

Each volume is complete for the year prior to its publication, on the subject of which it treats. It is published for the general practitioner and is published in several volumes, so that one may get the book on any special subject, and not be compelled to buy the whole set or a treatise on medicine and surgery. Price, per volume, $1.00, or $5.50 for the set of ten; payable in advance. We have before us:

THERAPEUTICS—PREVENTIVE MEDICINE—CLIMATOLOGY—FORENSIC MEDICINE. July, 1904.

PHYSIOLOGY—PATHOLOGY—BACTERIOLOGY. August, 1904.

SKIN —VENEREAL DISEASE — NERVOUS — MENTAL. September, 1904.

---

SURGICAL EMERGENCIES—THE SURGERY OF THE ABDOMEN. A Text-Book of Surgery for Practitioners of Medicine, Part 1. Appendicitis and Other Disease About the Appendix. By Bayard Holmes, M. D., Professor of Surgery, University of Illinois, etc. New York: D. Appleton & Co., 1904. Paper; 330 pages. Price not stated. Illustrated copiously.

This embodies "the author's experience as corrected by the experience of other operators in the surgery of the most mournfully interesting and unique structure in the body." He says: "This little retrogressive organ is responsible for an unusual death-rate among young and vigorous adults, and it breeds for others a series of nutritional and nervous disorders which made it worthy of the consideration of every practicing physician.

---

ANNUAL REPORT OF THE SURGEON GENERAL OF THE PUBLIC HEALTH AND MARINE HOSPITAL SERVICE OF THE UNITED STATES FOR 1904. Washington: Government Printing Office.

This report is of unusual interest to the Texas profession because of the space given to a "Report of the Epidemic of Yellow Fever of 1903 at Laredo, Minera and Cannel, Texas," by Marine Hospital Service Surgeon G. M. Guiteras, and the work of the Texas Public Health Service. Texas has reason to be very proud of her State Health Officer and his creditable work. The day is here now, when stopping the running of cars will be no longer necessary. Thanks to the lessons learned from a study of that epidemic.

---

THE MEDICAL NEWS VISITING LIST FOR 1905.—Lea Brothers & Co., New York and Philadelphia. Nineteenth year of issue. An invaluable, pocket-size, wallet-shaped book, containing memoranda and data important to every physician, and ruled blanks for recording every detail of practice. Price, $1.25.

The Perpetual Visiting List and Pocket Reference Book—Including information in emergencies, from standard authors. St. Louis: Dio Chemical Co., 1905. Sent on receipt of 10 cents. "Red Back."

---

Quiz Companies—Medical Latin, St. Clair. Blakiston's Sons & Co. Philadelphia. Cloth, $1.00.

Designed expressly for elementary training of medical students. Second edition, revised. Aim is to present to the medical student in a plain and practical way the fundamental principles upon which the medical language is built. I think some *old physicians* who speak of the "iodide of potash" and the "carbonate of creosote," and who put Latin terminals to English stems would do well to study these "elementary principles."

---

A Compend of the Practice of Medicine. By Daniel E. Hughes, M. D., Late Chief Resident Physician, Philadelphia Hospital, etc. Seventh revised edition. Edited, revised and partly rewritten by Samuel Norton Brown, M. D., Assistant Dermatologist, Philadelphia Hospital, etc. Including a Section on Skin Diseases. Philadelphia: P. Blakiston's Sons & Co.

Flexible cover of fine Morocco, gilt edges. 746 pages (looks like a Bible). Nice paper, clear print. Price, $2.50.

This is one of the dandiest books in its get-up we have seen. It is a luxury to own such a copy. For ready reference it is just the thing to obviate going through a large work on Practice. In the revision, 137 pages have been added, and 27 illustrations have been inserted. New remedies and modes have been added to bring it up to date. An unusually comprehensive table of contents is a feature that will facilitate reference to any subject sought. There are lots of things in this edition not in the earlier ones.

---

THE SURGICAL TREATMENT OF BRIGHT'S DISEASE. By Geo. M. Edelbohls, A. M., M. D., LL. D., Professor of Diseases of Women in the New York Post-Graduate Medical School and Hospital, etc. Frank F. Lisiecke, Publisher, 9 to 15 Murray Street, New York. Price not stated. Cloth and gilt; fine paper; 320 pages. 1904.

The author says: "There is * * * a very active and insistent demand on the part of the medical profession for such facts and information, especially as regards results, as may at present be available concerning the new treatment of so common and so fatal a malady as chronic nephritis. To meet this demand as far as it is possible at the present writing is the object of this volume." The book is made up in large part of the author's contributions to the medical press, some, very recently; the larger part of the text being entirely new matter never before published, and dealing almost wholly with results. Every doctor will want to read this book, for the subject of Bright's Disease is one about which very little is really known; i. e., most cases die.

---

THE UPS AND DOWNS OF A VIRGINIA DOCTOR. By C. A. Brice, M. D., Richmond, Va., Editor and Publisher of *The Southern Clinic.* Illustrated from drawings by Mildred Brice, Publisher and Editor of "The Hoppergrass." Cloth, $1.00.

This is about the best dollar's worth on the market. The funny little drawings by Dr. Brice's daughter are worth a dollar each of any doctor's wealth, who has any kind of sense or appreciation. Reading the book is a luxury. Mrs. D. and I read it twice. The only objection *we* had to it was that—like the little boy's candy—there was not enough of it. Ah! Brice hits some of the hypocrites and would-be-great fellows some center shots that must give them the blind-staggers. His case is one of "be honest and be poor." Brice is a trump—a free-lance that goes for shenanegan in a straight line, and gets there. *Of course,* he has enemies.

All honest men have enemies. I wish all the Texas doctors would read Brice's book. It's more than "interesting"; it's wholesome, and an eye-opener.

---

QUALITATIVE ANALYSIS BRIEF.—Allard Memminger, M. D., Professor of Chemistry, Hygiene and Clinical Urinary Analysis in the Medical College of South Carolina, etc. Blakiston's Sons & Co., Philadelphia. 1904. Second edition, revised and rewritten. Cloth, 110 pages, $1.00.

This is a handy and authoritative manual to guide the student in qualitative work in the laboratory examination of urine. It will facilitate the work very much.

